# Psychological Distress in the Hospital Setting: A Comparison between Native Dutch and Immigrant Patients

**DOI:** 10.1371/journal.pone.0130961

**Published:** 2015-06-25

**Authors:** Gertrud L. G. Haverkamp, Bart Torensma, Anton C. M. Vergouwen, Adriaan Honig

**Affiliations:** 1 Department of Psychiatry, Sint Lucas Andreas Hospital, Amsterdam, The Netherlands; 2 Research department, Sint Lucas Andreas Hospital, Amsterdam, The Netherlands; 3 Department of psychiatry, VU Medical Centre, Amsterdam, The Netherlands; Queensland University of Technology, AUSTRALIA

## Abstract

**Background:**

Prevalence of psychological distress (i.e. depressive and anxiety symptoms) in medically ill patients is high. Research in the general population shows a higher prevalence of psychological distress among immigrants compared to natives. Our aim was to examine the prevalence of psychological distress in the hospital setting comparing immigrant and native Dutch patients and first and second generation immigrant patients.

**Methods:**

Prevalence of psychological distress was assessed using the extended Kessler-10 (EK-10) in 904 patients in a Dutch general teaching hospital. Logistic regression was used to calculate odds ratios to determine differences between native and immigrant patients and first and second generation immigrants in the prevalence of psychological distress. We adjusted for demographic and social variables, socio-economic status, physical quality of life, history of psychiatric disease and health care use.

**Results:**

Of 904 patients, 585 were native Dutch patients and 319 were immigrant patients. The prevalence of psychological distress in native compared to immigrant patients was 54% and 66% respectively, with especially high prevalences among Turkish and Moroccan immigrant patients. The crude OR for prevalence of psychological distress for immigrant patients versus native patients was 1.7 (95% CI 1.2–2.2) and for first versus second generation immigrant patients 2.1 (95% CI 1.2–3.5). After full adjustment ORs were 1.7 (95% CI 1.2–2.3) and 2.2 (95% CI 1.2–4.1) respectively.

**Conclusion:**

Immigrant patients and first generation immigrant patients were more likely to have psychological distress compared to native patients and second generation immigrant patients respectively. We found a particularly high prevalence of psychological distress in Turkish and Moroccan immigrants.

## Introduction

Psychological distress (i.e. depressive- and anxiety symptoms) is a well-known risk factor for an adverse course of medical illness and is associated with impairment of quality of life, increased risk of mortality and significantly higher health care costs [1–3]. Prevalence estimates for depressive and anxiety symptoms in the medically ill are high compared to the general population and range from 14%-51% and 18%-32% respectively [1–4].

Besides the high prevalence of depressive and anxiety symptoms in the medically ill, previous research in the general population also shows a higher prevalence among immigrants compared to natives [5–7]. Migration in Europe has been high during the last decades and the major immigrant groups are from Turkey and Morocco [8]. Turkish and Moroccan men came in the mid-1960s as guest workers to Europe, followed by their families in the 70s [9]. A study in Amsterdam showed a prevalence of depression of 18.7% in Turkish respondents, 9.8% in Moroccan, and 6.6% in Dutch respondents [5]. Previous studies in Europe also found a higher prevalence of depressive symptoms among first compared to second generation immigrants [7,10]. More significant losses in the country of origin (e.g. social contacts, loss of profession) and higher acculturation stress (adaptation to the new country) in first generation immigrants may be responsible for this difference[10]. However, this difference in immigrant generation is less often studied, as second generation immigrants are, according to the country of birth, most often seen as natives [7].

So far, most of the research has focused on depressive and anxiety symptoms of immigrants in the general population [5–7], in primary care [11], or in mental health care [12] and focused on other mental disorders as psychotic disorders [13]. We found no studies examining the prevalence of depressive and anxiety symptoms in immigrants in the general hospital setting and it is not clear whether the prevalence of psychological distress in the hospital setting is also higher among immigrant patients and to which extent compared to native patients.

The aim of this study is to examine the prevalence of psychological distress in the hospital setting comparing immigrant and native Dutch patients and first and second generation immigrants. Our hypothesis is that the prevalence of psychological distress will be higher among immigrant patients and first generation immigrants. Furthermore, we examined whether possible disparities between immigrant and native Dutch patients and first and second generation immigrants can be explained by demographic and social variables, socio-economic status, physical quality of life, history of psychiatric disease and health care use.

## Material and Methods

### Study design

This cross-sectional study was conducted in a general teaching hospital in Amsterdam West, the Netherlands, in the period October 2011 to January 2012. The catchment area consists of 160.000 residents, of which 47% are of non-Western origin, according to the Research and Statistics, City of Amsterdam [14]. In 2011 the hospital recorded almost 28.000 clinical admissions, 18.000 day treatments and 112.000 first outpatient visits. The study was approved by the Medical Ethics Committee of the Sint Lucas Andreas hospital (Approval number: mec11/096 SOMA en PSYCHE) and all patients included gave written informed consent.

Patients were approached to complete a questionnaire (specified in the measures section) in all hospital settings, namely inpatients, outpatients and day therapy. All departments were visited, except the psychiatric and paediatric departments. We included patients in the following clinical and outpatient departments: surgery, internal medicine, gynaecology, otolaryngology, plastic surgery, urology, orthopaedics, neurosurgery, oncology, neurology, cardiology, pulmonology, and gastroenterology. As a result, patients with a broad range of conditions are included.

Patients could be enrolled in only one department. Inclusion took place in three separate rounds. The first two rounds of inclusion all participating departments were visited and during the third round the departments with a relatively small number of included patients were revisited in order to create an as equally distributed sample as possible.

Patients were included in case they were ≥ 18 years of age and were treated in one of the surgical or medical departments of the hospital or being treated and having an appointment in an outpatient setting. Exclusion criteria were: severe cognitive impairment or presence of a confusional state, patients physically too ill to participate, isolation treatment and illiteracy or insufficient knowledge of the Dutch language to complete the questionnaire with help from the researchers.

### Measures

We collected the following data from electronic medical records: age, gender, marital status, having children, socio-economic status and health care use. The socio-economic status was defined low or normal by tracing the zip code area were the patients resided. Zip code areas classified as disadvantaged neighbourhood is determined nationwide, based on, for example, population density, educational level, and percentage of low income [15]. Finally, to examine health care use we noted the number of visits to outpatient clinics and admissions in the year prior to participation.

Ethnicity was determined based on self-reported country of birth of patients and their parents. We made a distinction in native patients and immigrant patients. According to the Statistics Netherlands criteria [16], an immigrant is defined as an individual of whom at least one parent was born abroad, regardless of the own country of birth. Furthermore, we made a distinction in first generation and second generation immigrant patients. First generation immigrants are patients who are born abroad with at least one parent also born abroad. Second generation immigrants are patients born in The Netherlands with at least one parent belonging to the first generation.

We used the Dutch version of the Extended Kessler-10 (EK-10), a short questionnaire that screens broadly for psychological distress (depressive- and/or anxiety symptoms). The EK-10 consists of the Kessler-10 (K-10) [17], extended with five additional questions focusing on anxiety symptoms. This scale was chosen, because it was recently validated in the primary care setting in the Netherlands (including Amsterdam) [18], in patient groups with comparable demographic characteristics to our participants. It was also chosen because of the strong psychometric properties and its ability to discriminate DSM-IV disorders from non-cases. The EK-10 has a sensitivity of 0.90 and specificity of 0.75 for detecting any depressive and/or anxiety disorder when a cut-off point of 20 is used for the first 10 questions and/or at least one positive answer on the additional questions [18].

Health related quality of life was assessed using the Short-Form 12 Health Survey (SF-12) [19]. The SF-12 is a 12 item survey comprising two summary measures, the physical component summary (PCS) and the mental component summary (MCS) [20]. We only used the PCS, because of overlap in measurement of the MCS and EK-10. A lower score on the PCS is indicating a poorer physical health.

Additionally, participants completed questions to assess their history of psychiatric disorders and to define the hospital setting and medical specialism.

### Data analysis

All data were first tested for normality by a Kolmogorov-Smirnov test, a Q-Q plot and Levene’s test.

The difference between included and excluded cases on the basis of age and gender was analysed to detect possible selection bias and descriptive statistics was used to outline the characteristics of the patients. Categorical variables were expressed as n (%). Continuous normally distributed variables were expressed by their mean and standard deviation, not normally distributed data by their median and interquartile range for skewed distributions.

To test groups, categorical variables were tested using the Pearson’s Chi-square test. Normally distributed continuous data were tested with the independent samples Students t-test and in case of skewed data, with the independent samples Mann-Whitney U-test. Logistic regression was used to calculate odds ratios to determine differences between native and immigrant patients and first and second generation immigrants in the prevalence of psychological distress. Multivariable adjustment was done deliberately within the causal pathway between ethnicity and psychological distress, in order to investigate whether possible differences between immigrant and native patients are a consequence of certain variables. We adjusted for demographic and social variables, socio-economic status, physical quality of life, history of psychiatric disease and health care use.

Up to 2 missing values on the K-10 were imputed through replacement by individual mean item score, based on validation studies in The Netherlands [18]. Up to three missing values on the SF-12 were imputed by predicting the missing item weight from the sum of the other non-missing items weights [21]. If there were more missing values than described above the patient was not included in the data analysis. Analysis were performed in SPSS 21.0 for Windows statistical software. Significance level for baseline variables and univariate and multivariate regression analysis was set at p value ≤ 0.05.

## Results

### Patient sample

Of the 1150 patients eligible for study participation, 936 patients (81%) agreed to participate. Thirty-two patients were excluded because of too many missing values on the EK-10 or SF-12 for imputation or missings in the ethnicity related questions. Leaving 904 participating patients, of which 585 (65%) native patients and 319 immigrant patients (35%). Patients who refused to participate did not significantly differ from the included sample in mean age (60 years and 58 years respectively) and gender (40% and 46% male respectively).

The baseline characteristics of the 904 participating patients are shown in Table 1. Seventy-seven percent of the immigrant patients were first generation immigrants. The native patients were significantly older, less often had children and less often had a low socio-economic status compared to the

immigrant patients.

**Table 1:** Baseline characteristics of the native and immigrant patients.

	Total N = 904	Natives^1^ N = 585	Immigrants^2^ N = 319	P-value
Age mean (±SD)	58 (18)	61 (17)	51 (16)	0.00*
Gender				
Male, *%*	46	47	45	0.47**
Ethnicity				
Native Dutch^1^, N (%)	585 (65)	**-**	**-**	**-**
Moroccan^2^, N (%)	51 (6)	**-**	51 (16)	**-**
Surinamese^2^, N (%)	63 (7)	-	63 (20)	**-**
Indonesian^2^, N (%)	35 (4)	**-**	35 (11)	**-**
Turkish^2^, N (%)	56 (6)	**-**	56 (18)	**-**
Remaining^2^, N (%)	114 (12)	-	114 (35)	**-**
First generation immigrants, N (%)	244 (27)	***-***	244 (77)	**-**
Married/living together, yes %	53	53	52	0.48**
Having children, yes %	56	52	62	0.01**
Low socio-economic status, yes %	38	31	51	0.00**
History of psychiatric disorders, yes %	27	25	30	0.12**
Physical Component Summary, mean (±SD)	42 (10)	41 (10)	42 (9)	0.13**
Hospital setting				
Inpatients, N (%)	301 (33)	196 (34)	105 (33)	0.86**
Outpatients and day therapy, N (%)	603 (67)	389 (66)	214 (67)	
Specialism				
Surgery, N (%)	449 (50)	287 (49)	162 (51)	0.62**
Non-surgery, N (%)	455 (50)	298 (51)	157 (49)	
Visits to outpatient clinics, median (range)	6 (0–81)	6 (0–81)	7 (0–61)	0.58***
Number of admissions, median (range)	1 (0–15)	1 (0–15)	1 (0–10)	0.91***

^1^Patient born in the Netherlands and both parents born in the Netherlands

^2^ One or both parents born abroad

* Unpaired sample T-test

** Chi square test

*** Mann-Whitney U-test

### Prevalence of psychological distress

The overall prevalence of psychological distress found was 58% (Table 2). Psychological distress was not significantly related to age or gender. We found significant associations between ethnicity and psychological distress. In the native Dutch patients a 54% prevalence of psychological distress was found and in the immigrant patient group a 66% presence (p = 0.00). The prevalence was highest among Turkish and Moroccan patients, respectively 79% and 73%. In Indonesian and Surinamese patients the prevalence of distress is more similar to native patients. When comparing first and second generation immigrant patients a significantly higher prevalence of psychological distress was found in first generation immigrants, 70% versus 53% respectively. Depressive and anxiety symptoms analysed separately, using the K10 questions to measure depressive symptoms and the five extended questions to measure anxiety symptoms, showed significantly higher prevalences of both depressive and anxiety symptoms among immigrant patients compared to native Dutch patients, 58.6% versus 45.6% (p≤0.01) and 51.7% versus 36.6% (p≤0.01) respectively (data not shown).

**Table 2:** Association of presence of absence of psychological distress with age, gender, ethnicity, immigrant status, quality of life, setting and specialism.

	Psychological distress N = 528 (58%)	No Psychological distress N = 376 (42%)	P value
Age mean (±SD)	58 (17)	57 (18)	0.56*
Gender			
Male, N (%)	235 (56)	182 (44)	0.25**
Female, N (%)	293 (60)	194 (40)	
Ethnicity			
Dutch nationals, N (%)	317 (54)	268 (46)	0.00 **
Immigrants, N (%)	211 (66)	108 (34)	
Origin			
Moroccan, N (%)	37 (73)	14 (27)	-
Surinamese, N (%)	36 (57)	27 (43)	-
Indonesian, N (%)	16 (46)	19 (54)	-
Turkish, N (%)	44 (79)	12 (21)	-
Remaining, N (%)	78 (68)	36 (32)	-
Immigrant status			
First generation immigrants, N (%)	171 (70)	73 (30)	0.01**
Second generation immigrants, N (%)	40 (53)	35 (47)	
Quality of life			
Physical Component Summary, mean (±SD)	39.8 (9)	44.3 (9)	0.00 **
Setting			
Inpatients, N (%)	182 (60)	119 (40)	0.38**
Outpatients and day therapy, N (%)	346 (57)	257 (43)	
Specialism			
Surgery, N (%)	247 (55)	202 (45)	0.04**
Non-surgery, N (%)	281 (62)	174 (38)	

* T-Test

** Chi square test

We found no significant difference in the prevalence of psychological distress in the various hospital settings (p = 0.38). In the inpatient and outpatient/day therapy setting the prevalence of psychological distress was 60% and 57% respectively. When comparing surgical and non-surgical disciplines, we found a significant difference in the prevalence of distress (p = 0.04), with a prevalence of 62% in non-surgical disciplines and 55% in surgical disciplines.

### Regression analysis of presence of psychological distress

The crude OR for prevalence of psychological distress for immigrant patients versus native patients was 1.7 (95% CI 1.2–2.2) and for first versus second generation immigrant patients 2.1 (95% CI 1.2–3.5). After adjustment for demographic variables, social variables and socio-economic status, ORs were 1.6 (95% CI 1.2–2.2) and 2.0 (1.2–3.6) respectively. Further adjustment for physical quality of life, history of psychiatric disease and health care use did not influence the ORs (Models 5–7, Table 3). We found no differences comparing second generation immigrant patients and native patients (data not shown in table).

**Table 3:** Odds ratios for presence of psychological distress for immigrant patients versus native patients and first generation immigrant patients versus second generation immigrant patients.

		Immigrants versus natives	First versus second generation immigrants
Model	Variables tested	OR (95% CI)	OR (95% CI)
1. Unadjusted	Ethnicity	1.7 (1.2–2.2)	2.1 (1.2–3.5)
2. Demographic variables	Model 1 plus age, gender	1.8 (1.3–2.4)	2.1 (1.2–3.7)
3. Social variables	Model 2 plus marital status, children	1.7 (1.3–2.3)	2.2 (1.2–3.8)
4. Socio-economic status	Model 3 plus socio-economic status	1.6 (1.2–2.2)	2.0 (1.2–3.6)
5. Quality of life	Model 4 plus PCS score	1.6 (1.2–2.2)	2.4 (1.3–4.3)
6. History of psychiatric disease	Model 5 plus history of psychiatric disease	1.7 (1.2–2.3)	2.2 (1.2–4.1)
7. Health care use	Model 6 plus visits to outpatient clinics and number of admissions	1.7 (1.2–2.3)	2.2 (1.2–4.1)

## Discussion

This cross-sectional study examined the difference in prevalence of psychological distress in immigrant and native patients and first and second generation immigrant patients in a general teaching hospital. We found a high overall prevalence rate of psychological distress of 58%. Immigrant patients were 1.7 times more likely to have psychological distress compared to native patients, also after adjustment for several risk factors. The prevalence rate of psychological distress was especially high in Turkish and Moroccan patients. The OR for prevalence of psychological distress for first compared to second generation immigrants was 2.1, and 2.2 after adjustment for several risk factors.

The overall prevalence rate of psychological distress in this study is high. The use of various screening lists and different cut-off values in research creates a wide range of prevalences of depressive and anxiety symptoms. Comparable to our study is a study on the prevalence of psychological distress (measured with the EK-10) in the primary care setting in The Netherlands [22]. That study found a prevalence of psychological distress of 43%. An explanation for the difference in prevalence is probably that patients in the primary care setting are less ill than those in the general hospital, who face more stressful experiences such as invasive testing and possibility of an uncertain diagnosis [23,24].

Regarding the higher prevalence of psychological distress among immigrant compared to native patients our results are in line with previous literature in the European general population [5–7,25]. An important aforementioned explanation for this difference is the lower socio-economic status of immigrants [26]. Despite that we also more often found a lower socio-economic status among immigrant patients, we did not find a clear effect of socio-economic status in the regression analysis. Also in the study of De Wit et al [5], socio-economic differences could not explain the ethnic differences found. Other frequently mentioned explanations are discrimination and racism, recent immigration and acculturative stress [7,27–29]. Especially in the hospital setting, a language barrier and as a result difficulties concerning the communication about the disease, may increase distress in immigrant patients [30].

Also the higher prevalence among Turkish and Moroccan immigrant patients compared to other immigrants is consistent with other European studies in the general population [5,6]. A possible explanation could be that the prevalences are also higher in their native countries. However, an international survey in ten countries, showed a lifetime prevalence of depression significantly higher in the Netherlands compared to Turkey, 15.7% and 6.3% respectively [31]. On the other hand, a study in Turkey among inpatients showed a prevalence of depressive symptoms of 77.4% [32], which is very high. In Morocco, a study among a representative sample of the general population also showed a very high prevalence of depression and anxiety of 26.5% and 37% respectively [33]. Another explanation could be that the Turkish and Moroccan immigrants are Muslim, whereas the other immigrant groups do not consist of Muslims or a much smaller proportion. In recent years the position of Muslim immigrants in Dutch society became more stressful, this might also contribute to a decreased mental health [34].

We found a higher prevalence of psychological distress in first compared to second generation immigrant patients, which is also in line with previous studies in Europe [7,10]. Furthermore, we found no differences between second generation immigrant patients and native patients. This could mean that the role of the migration process (e.g. migration stress, acculturation) on the development of psychological distress is the most important [7,35], however, we did not examine this. We also cannot conclude whether the first generation immigrants developed the psychological distress in the country of origin or after migration. Most immigrants in this study are from Turkey, Morocco, Surinam and Indonesia. Immigrants from the first two countries came as guest workers and from the latter two from former colonies [9]. Most of these may have migrated to the Netherlands not because of political persecution but to improve their economic opportunities.

There are some study limitations: first, the socio-economic status of the patients was defined using the zip code areas. As areas are defined as a disadvantaged neighbourhood based on for example educational level and percentage of low income [15], it approximates the actual socio-economic status of patients sufficiently. However, we do not know exactly the individual level of education, employment status or income. Second, we do not have any information on the length of stay in The Netherlands of the first generation immigrant patients, reasons for immigration and experience of discrimination. These factors all may influence the higher prevalence of psychological distress in the immigrant patients. A third limitation is that we had to exclude 101 patients because of a language barrier and therefore they are probably first generation immigrant patients. Kamperman et al [30] shows that communication barriers negatively affect psychological well-being and thus these participants are more at risk of distress. It is therefore plausible that the percentage of psychological distress in the first generation immigrant patient group is underestimated in our study. We cannot report about the remaining immigrant patient group, because this group contains patients from Western and non-Western countries and these immigrant patient groups would be very small. Finally, it has to be considered that the results found on the EK-10 in groups with different ethnicity may be subject to cultural bias [36] as the interpretation of the questions might have been different among different ethnic groups [37] and the symptomatic expression of depression and anxiety may vary [38].

In conclusion, the overall prevalence of psychological distress in the hospital setting was high. Immigrant patients were, after adjustment for several risk factors, 1.7 times more likely to have psychological distress compared to native patients. We found a particularly high prevalence of psychological distress in Turkish and Moroccan immigrant patients. First generation immigrant patients were 2.2 times more likely to have psychological distress compared to second generation immigrant patients after adjustment for several risk factors. As psychological distress is a risk factor for an adverse course of medical illness, increased mortality, and higher health care consumption, it is important that clinicians in all hospital settings, recognize psychological distress by standard screening and subsequently provide appropriate care or referral [39]. Since migration in Europe continues to increase, clinicians should be aware that immigrant patients, especially first generation immigrant patients, are at a higher risk of developing psychological distress compared to native patients. Future research may focus on other risk factors that may explain the differences found, for example experience of discrimination and acculturation.
