# Correction: Psychological Distress in the Hospital Setting: A Comparison between Native Dutch and Immigrant Patients

**DOI:** 10.1371/journal.pone.0159697

**Published:** 2016-07-18

**Authors:** Gertrud L. G. Haverkamp, Bart Torensma, Anton C. M. Vergouwen, Adriaan Honig

The Data Availability statement for this paper is incorrect. The correct statement is: Data are available from Figshare at https://dx.doi.org/10.6084/m9.figshare.3462608.v1.
